# The millipede genus *Tetracentrosternus* Pocock, 1895 (Polydesmida, Paradoxosomatidae, Alogolykinae, Alogolykini), with a description of the first, new species from Thailand

**DOI:** 10.3897/zookeys.358.6582

**Published:** 2013-12-03

**Authors:** Natdanai Likhitrakarn, Sergei I. Golovatch, Somsak Panha

**Affiliations:** 1 Animal Systematics Research Unit, Department of Biology, Faculty of Science, Chulalongkorn University, Bangkok, 10330, Thailand; 2 Institute for Problems of Ecology and Evolution, Russian Academy of Sciences, Leninsky pr. 33, Moscow 119071, Russia

**Keywords:** Millipede, Alogolykinae, Alogolykini, *Tetracentrosternus*, taxonomy, new species, Thailand

## Abstract

The millipede subfamily Alogolykinae and the tribe Alogolykini are recorded in Thailand for the first time,being represented there by *Tetracentrosternus theelorsuensis* **sp. n.** While the new species has characteristics that place it in the genus *Tetracentrosternus*, it also shows a number of unique features that make it necessary to rediagnose this Oriental genus, as well as to key its three currently known species and map their distributions. The tribes Alogolykini and Polydrepanini, as well as the subfamily Alogolykinae are also briefly redefined.

## Introduction

The family Paradoxosomatidae has long been known to dominate the millipede fauna of the Oriental realm, including Thailand (Jeekel 1968). Thus, this family accounts for about half of the country’s known diplopod species richness (Enghoff 2005). According to our latest estimates, Thailand currently supports 161 nominal species of Diplopoda, of which 71 (about 44%) are paradoxosomatids. They all belong to the nominotypical subfamily Paradoxosomatinae.

All the more interesting is the discovery in Thailand of the first species of the small, purely Oriental subfamily Alogolykinae. This new species, belonging in the small genus *Tetracentrosternus* Pocock, 1895 from the tribe Alogolykini, is described below. Several of its characters are so peculiar that the genus requires a redefinition.

Only two species of *Tetracentrosternus* have hitherto been known: *Tetracentrosternus subspinosus* Pocock, 1895, the type species from Myanmar (Pocock 1895, Hoffman 1963, Jeekel 1965), and *Tetracentrosternus hoffmani* Golovatch, 2013, from Yunnan, southern China (Golovatch 2013).

## Material and methods

The material was collected during a field trip in January 2011 from near Thee Lor Sue Waterfall in the mountains at the frontier between Thailand and Myanmar. Live animals were photographed on the spot. Specimens were preserved in 75% ethanol and studied in the laboratory using an Olympus stereomicroscope. Scanning electron micrographs (SEM) of the right gonopod coated with gold were taken using a JEOL, JSM–5410 LV microscope, returned to alcohol upon examination. Digital images of the specimen were taken in the laboratory and assembled using the “Cell^D^” automontage software of the Olympus Soft Imaging Solution GmbH package. In addition, line drawings of the left, completely intact gonopod were also prepared. All material is housed in the Museum of Zoology, Chulalongkorn University (CUMZ), Bangkok, Thailand.

## Systematics

### Family Paradoxosomatidae Daday, 1889
Subfamily Alogolykinae Hoffman, 1963
Tribe Alogolykini Hoffman, 1963

#### Genus Tetracentrosternus

Pocock, 1895

http://species-id.net/wiki/Tetracentrosternus

##### Diagnosis.

Body medium-sized (ca 20–30 mm long, ca 2.0–3.2 mm wide), with 20 segments. Paraterga moderately to strongly developed. Sterna unmodified or with a cone near each coxa. Sternal lobe or cone(s) between ♂ coxae 4 present. First pair of ♂ legs with or without femoral adenostyles. At least some male legs with ventral brushes on tarsi, sometimes also on distal halves of tibiae.

Gonopod with a quite short, subcylindrical, distoventrally densely setose coxite; prefemoral (= setose) part of telopodite short to very short, a quarter or less as long as acropodite, delimited from extremely short femorite by a mesal sulcus, ridge or spine; femorite supporting a long, spiniform, sometimes deeply bipartite femoral process (**fp**) and a similarly long to longer, flagelliform solenomere (**sl**), both more or less clearly sheathed by a mesal fold in basal half of acropodite. The latter suberect to strongly unciform, distal quarter to third with or without evident processes, clearly fimbriate and/or fringed, often phylloid as well.

##### Type species.

*Tetracentrosternus subspinosus* Pocock, 1895; by monotypy.

Other species include *Tetracentrosternus hoffmani* Golovatch, 2013 and *Tetracentrosternus theelorsuensis* sp. n.

**Remarks.** Both *Tetracentrosternus* Pocock, 1895 and *Tetracentrosternus subspinosus* Pocock, 1895 were originally diagnosed and described too briefly to be readily recognizable. The species was based on a few specimens taken by L. Fea and E. W. Oates at Puepoli (900–1,200 m a.s.l.) and Bia-po, now Carin Cheba (1,000–1,200 m), both in Myanmar (Pocock 1895, Thorell 1895). These two localities lie in the Karin Hills of southeastern Myanmar, in the Tenasserim Mountain Range adjacent to Thailand (Fig. 4).

The sole male of the species, from Puepoli and kept in the British Museum, was redescribed by Hoffman (1963) who designated it as lectotype. Hoffman was the first to provide clear illustrations of its gonopod structure, resulting in the establishment of a new tribe, Alogolykini, to encompass *Tetracentrosternus*, *Alogolykus* Attems, 1936 and *Touranella* Attems, 1937 (Attems 1936, 1937, 1938). Jeekel (1965) redescribed this species in more detail not only from the lectotype, but also from the paralectotypes (from Carin Cheba) still housed in the Genoa Museum. He made new, even more accurate illustrations and a highly detailed redescription, fully accepting the tribe and adding thereto also *Yuennannina* Attems, 1936. A little later, Jeekel (1968) elevated the tribe Alogolykini to the status of a subfamily, Alogolykinae, adding thereto a new tribe, Polydrepanini. Jeekel transferred *Touranella* from Alogolykini to Polydrepanini, because the genus lacks adenostyles on the femora of the male first legs, as do the other constituent genera of the latter tribe.

*Tetracentrosternus hoffmani* Golovatch, 2013 stems from Mount Gaoligong Shan, western Yunnan, China (Golovatch 2013). Like *Tetracentrosternus subspinosus*, it shows adenostyles on male femora 1, a suberect gonopod telopodite with a fringed/fimbriate/spiculate and phylloid apex, and a long, unipartite gonofemoral process.

The new species described below, despite its relative geographical proximity to *Tetracentrosternus subspinosus*, shows a number of characters so different that the diagnosis of the genus needs to be refined. Thus, because there are no adenostyles in femora 1 of the male of *Tetracentrosternus theelorsuensis* sp. n., this trait can be regarded as only species-specific not only in *Tetracentrosternus*, but in the entire subfamily Alogolykinae. So, following Hoffman (1963), it seems best to return *Touranella* (three species in Vietnam, one in Nepal) to Alogolykini, as its close similarities to *Tetracentrosternus* are apparent. The only meaningful differences lie in gonopod structure, the femoral portion in *Touranella* sometimes being a little longer, the solenomere a little thicker, suberect and rod-shaped rather than flagelliform, while the femoral process when present is considerably shorter (Golovatch 2009a, 2009b). In our opinion, a strong, rod-shaped solenomere versus a thin, flagelliform one remains the basic difference between the tribes Alogolykini and Polydrepanini, respectively. Thereby the subfamily Alogolykinae seems to be best characterized only by the absence of a clear-cut division of the solenophore or solenophore-like structure near/around the solenomere into a membranous *lamina medialis* and/or a similarly membranous *lamina lateralis*, in Polydrepanini often coupled with a twisted, helicoid course of the seminal groove. In addition, the gonofemorite in Alogolykinae is often strongly abbreviated while many species show adenostyles on the male first femora. The latter two traits are also characteristic of the subfamily Australiosomatinae, but the solenophore branch or branches remain free and never sheath a primitively long, strong and rod-like solenomere. A more detailed review of these subfamilies and their tribes (see Jeekel 1968) lies far beyond the scope of the present note.

The gonopod telopodite in *Tetracentrosternus theelorsuensis* sp. n. is strongly elongate and unciform, bearing three evident processes in the distal half, whereas the solenomere is particularly long and nearly as long as the solenophore, while the femoral process is also very long, but deeply bipartite. Besides this, as in *Tetracentrosternus subspinosus*, the strongly abbreviated gonofemoral part in the new species is delimited distally by a distinct ridge, as opposed to a strong spine in *Tetracentrosternus hoffmani*.

In terms of metatergal structure, *Tetracentrosternus theelorsuensis* sp. n. is somewhat intermediate between both congeners, the tegument being only moderately rugulose as opposed to nearly smooth in *Tetracentrosternus subspinosus* or rather clearly tuberculate in the rear halves of metaterga in *Tetracentrosternus hoffmani*.

There is little doubt that more species of *Tetracentrosternus* await discovery at least in and between eastern Myanmar and southern China, including Thailand (Fig. 4). The same certainly holds true for some other Alogolykini as well, e.g. *Touranella*.

#### Tetracentrosternus theelorsuensis sp. n.

http://zoobank.org/B30D3F12-C18D-4FFA-A891-46736B54CC7A

http://species-id.net/wiki/Tetracentrosternus_theelorsuensis

Figs 1 –3

##### Holotype

♂ (CUMZ), Thailand, Tak Province, Umphang District, Thee Lor Sue Waterfall, 590 m a.s.l., 15°55'38"N, 98°45'13"E (converted from GPS data), 19.01.2011, leg. N. Likhitrakarn.

##### Paratypes.

♂ (CUMZ), same District, Mokro Subdistrict, roadside, 1,168 m a.s.l., 16°14'14"N, 98°59'23"E, 20.01.2011, leg. N. Likhitrakarn. 5 ♀, 1 juv. (CUMZ), same District, Pa Wai Waterfall, 804 m a.s.l., 16°34'30"N, 98°50'3"E, 20.01.2011, leg. S. Panha, C. Sutcharit & N. Likhitrakarn.

##### Name.

After Thee Lor Sue Waterfall, the type locality, which is the largest and highest waterfall in Thailand.

##### Diagnosis.

Differs from congeners mainly by the first pair of ♂ legs lacking femoral adenostyles, coupled with unmodified sterna and the distal half of the gonopod telopodite being strongly curved, elongate and distally carrying three evident processes. See also Remarks above and Key below.

##### Description.

Length 21–23.5 (♂) or 19.5–22.5 mm (♀), width of midbody pro- and metazona 1.92–1.98 and 2.54–2.75 mm (♂) or 2.07-2.35 and 2.77–3.12 mm (♀), respectively.

**Figure 1.**
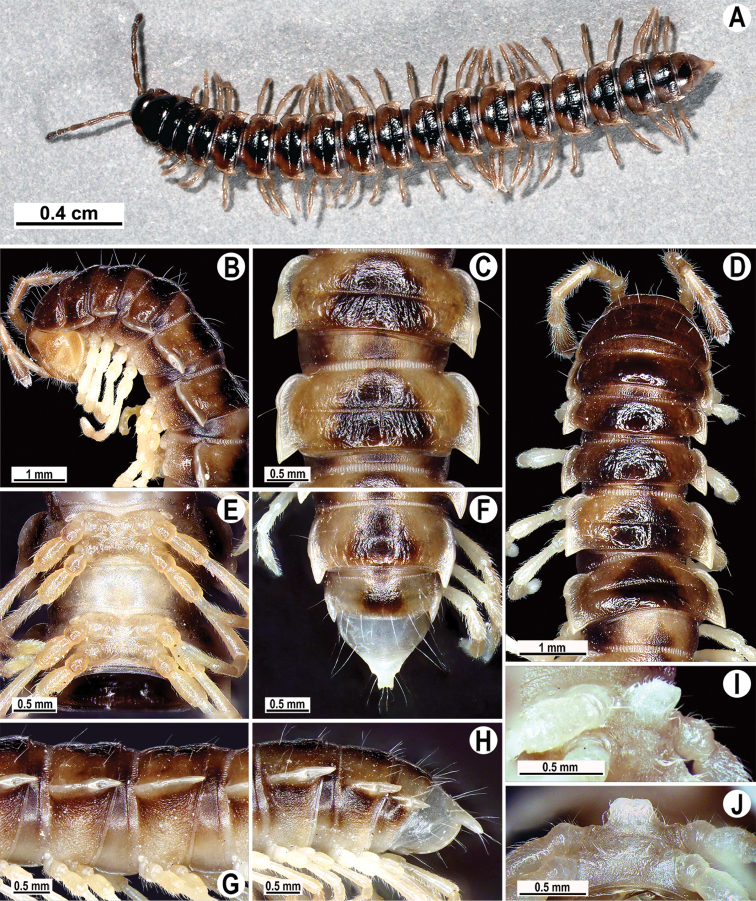
*Tetracentrosternus theelorsuensis* sp. n., ♂ holotype. **A** habitus, live coloration **B, D** anterior part of body, lateral and dorsal views, respectively **C, E, G** segments 10 and 11, dorsal, ventral and lateral views, respectively **F, H** posterior part of body, dorsal and lateral views, respectively **I, J** sternal lobe between coxae 4, sublateral and subcaudal views, respectively.

Coloration of live animals dark brown (Fig. 1A); paraterga, legs and epiproct light brown, head, collum and metazona 2–4 blackish, following terga with a blackish triangle covering both pro- and metazona; coloration of alcohol material after 2 years of preservation faded to whitish or yellow with a pattern of a contrasting dark brown triangle at posterior edge of pro- and metazona, castaneous brown below paraterga; head and antennomeres 6 and 7 brown to castaneous brown; venter and a few basal podomeres light brown to yellow-brown, legs growing infuscate (brown) distally; tip of antenna pallid (Fig. 1A–H).

Clypeolabral region and vertex sparsely setose, epicranial suture distinct. Antennae moderately long (Fig. 1A, B), reaching body segment 4 (♂) or 3 (♀) when stretched dorsally. In width, head < collum < segment 3 = 4 < 2 < 5–16 (♂, ♀), thereafter body gently and gradually tapering. Collum with three transverse rows of setae: 5+5 in anterior, 3+3 in intermediate and 4+4 in posterior row; a very faint incision laterally in posterior 1/3; caudal corner of paraterga rounded, slightly declined ventrad, not extending behind rear tergal margin (Fig. 1B, D). Tegument smooth and shining, prozona very finely shagreened, metaterga smooth and delicately rugulose, leathery; surface below paraterga finely microgranulate. Postcollum metaterga with two transverse rows of setae: 2+2 in anterior and 3+3 in posterior row, traceable at least as insertion points when setae broken off. Tergal setae long, strong and slender, about 1/3 of metatergal length. Axial line visible, barely traceable on proterga (♂). Paraterga strongly developed (Fig. 1A–H), especially so in ♂, mostly slightly upturned, lying high (at about upper third of midbody height); caudal corner nearly or fully pointed; either clearly (♂) or only very slightly extending beyond rear tergal margin (♀); paraterga very thin in lateral view, like blunt blades, a little thicker only on pore-bearing segments. Calluses on paraterga 2 delimited by a sulcus both dorsally and ventrally, on following paraterga only dorsally, rather broad on pore-bearing segments. Paraterga 2 broad, anterior edge angular, a little better so in ♀, lateral edge with two small, but evident incisions in anterior half; posterior edge clearly concave (Fig. 1B, D). Anterior edge of paraterga 3–9 clearly convex, of paraterga 10–18 nearly straight and slightly bordered. Lateral edge of paraterga with a slight, but evident incision in anterior 1/3. Posterior edge of paraterga clearly concave, especially strongly so in segments 16–19 (Fig. 1A–D). Ozopores evident, lateral, lying in an ovoid groove at about 1/4 in front of caudal corner. Transverse sulcus usually distinct (Fig. 1C, D, F), slightly incomplete on segments 4, 18 and 19, complete on metaterga 5–17, deep, not reaching bases of paraterga, at most faintly beaded at bottom, a little better developed in ♀. Stricture between pro- and metazona wide, evidently ribbed at bottom down to base of paraterga (Fig. 1B–D, F–H). Pleurosternal carinae complete crests with a sharp caudal tooth in segment 2, a small, caudal, mostly sharp tooth until segment 7 (♂) or 6 (♀) (Fig. 1B, G, H). Epiproct (Fig. 1F, H) conical, flattened dorsoventrally, with two small apical papillae; tip subtruncate; pre-apical papillae small, but visible, lying not too close to tip. Hypoproct roundly subtriangular, setiferous knobs at caudal edge well-separated and evident.

Sterna moderately setose, without modifications; a linguiform, sternal lobe between ♂ coxae 4 (Fig. 1I, J). Adenostyles absent on femur 1 (Fig. 3A). A paramedian pair of evident tubercles in front of gonopod aperture. Legs moderately long and slender, slightly incrassate in ♂, midbody ones ca 1.2–1.3 (♂) or 0.8–1.1 times (♀) as long as midbody height, prefemora without modifications, ♂ tarsal brushes present in all legs.

**Figure 2.**
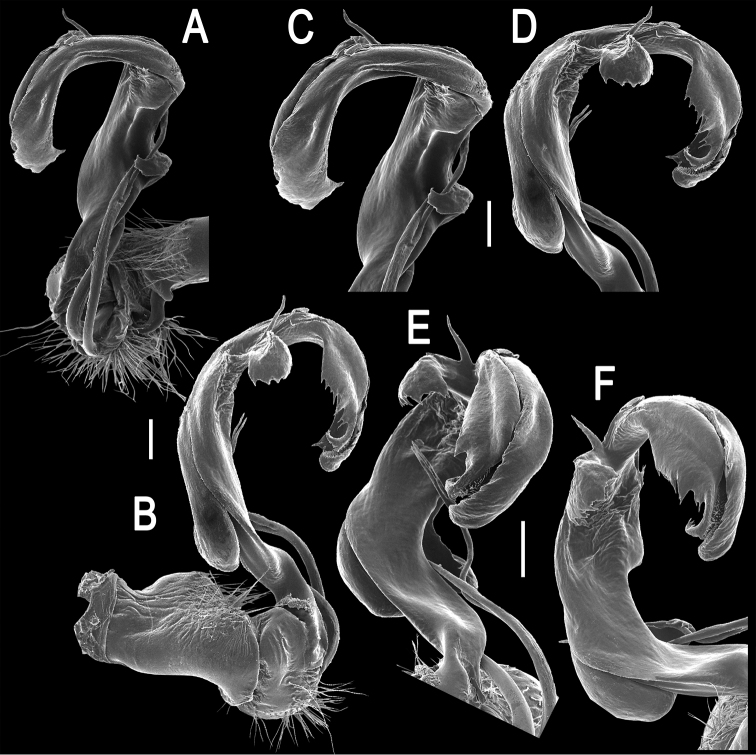
*Tetracentrosternus theelorsuensis* sp. n., ♂ holotype. **A, B** right gonopod (solenomere broken off), mesal and lateral views, respectively **C–F** distal part of right gonopod, mesal, lateral, suboral and subcaudal views, respectively. Scale bar: 0.2 mm.

**Figure 3.**
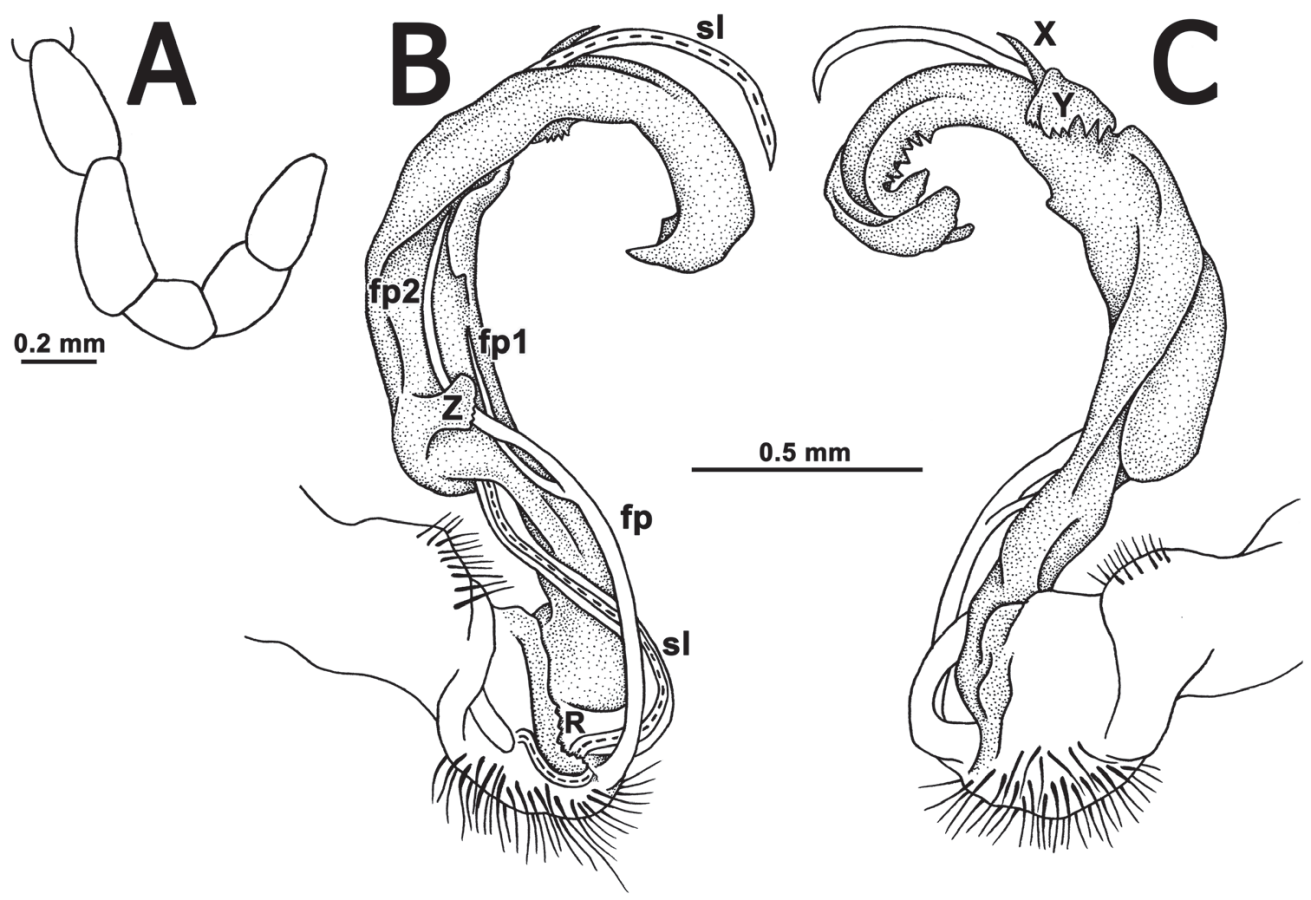
*Tetracentrosternus theelorsuensis* sp. n., ♂ holotype. **A** left first leg **B, C** left gonopod, mesal and lateral views, respectively.

Gonopods (Figs 2, 3B, C) complex. Coxa a little curved, short, subcylindrical, rather densely setose distoventrally. Prefemur densely setose, length less than 1/4 of femorite + “postfemoral” part. Femorite as usual, very short, delimited distally by a ridge (**R**). Femoral process (**fp**) a slender, distally deeply bipartite flagellum, with branch **fp2** somewhat longer than **fp1**. Acropodite (= solenophore) elongate, clearly unciform, in proximal 1/3 with a small, but distinct, mesal, apically denticulate process (**Z**), in distal 1/4 with a similar, but lateral and more strongly dentate process (**Y**), as well as a spine (**X**) at its base. Solenomere (**sl**) branching off level to **fp**, very long, flagelliform, mostly sheathed by a slightly longer solenophore.

**Figure 4.**
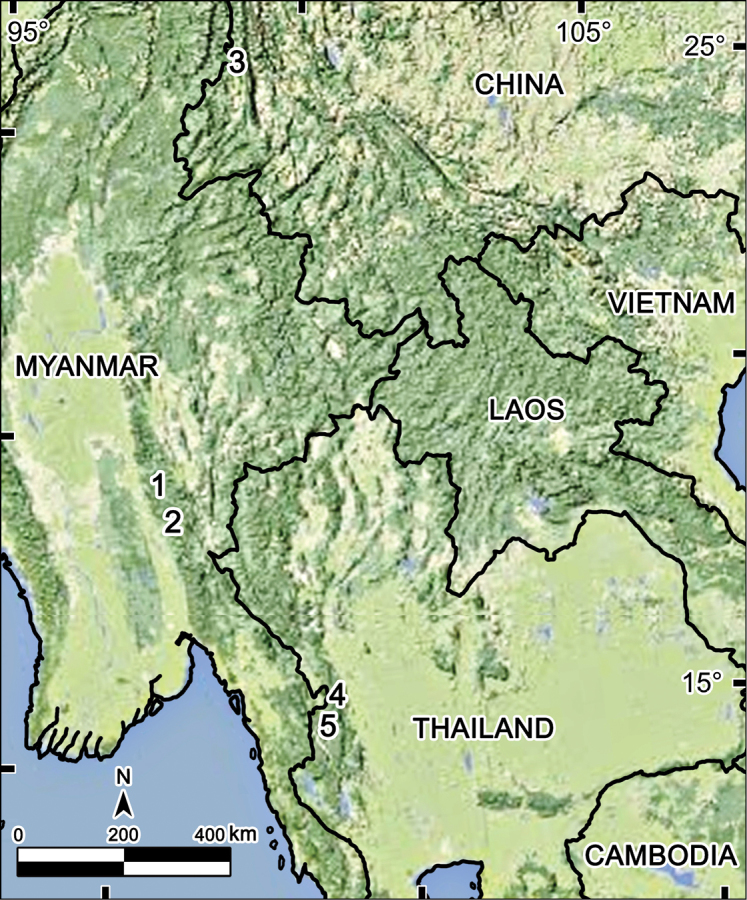
Distribution of *Tetracentrosternus* species. **1,2** *Tetracentrosternus subspinosus* (**1** Carin Cheba (= Bia-po) **2** Puepoli) **3** *Tetracentrosternus hoffmani* (Mt Gaoligong Shan) **4,5** *Tetracentrosternus theelorsuensis* sp. n. (**4** Thee Lor Sue Waterfall **5** Pa Wai Waterfall).

### Key to species of *Tetracentrosternus*

**Table:** 

1	Paraterga moderately developed, sternal cones present. Myanmar	*Tetracentrosternus subspinosus*
–	Paraterga strongly developed (Fig. 1A, C, D, F), sternal cones absent	2
2	Gonopod suberect, with a basal gonofemoral tooth and a distally strongly fimbriate/spiculate solenophore, but without evident processes distal to gonofemoral tooth. Southern China	*Tetracentrosternus hoffmani*
–	Gonopod strongly elongate and curved distally, supplied with three evident processes distal to femorite (Figs 2, 3). Thailand	*Tetracentrosternus theelorsuensis* sp. n.

## Supplementary Material

XML Treatment for Tetracentrosternus

XML Treatment for Tetracentrosternus theelorsuensis
